# Case Report: Catheter-associated multidrug-resistant *Leclercia adecarboxylata* bloodstream infection in a colon tumor patient

**DOI:** 10.3389/fmed.2026.1861083

**Published:** 2026-06-18

**Authors:** Ning Du, Dan You, Zhe Chen

**Affiliations:** 1 Department of Pharmacy, National Cancer Center/National Clinical Research Center for Cancer /Cancer Hospital & Shenzhen Hospital, Chinese Academy of Medical Sciences and Peking Union Medical College, Shenzhen, Guangdong, China; 2 Department of Pharmacy, The First Hospital of Qiqihar, Qiqihar, Heilongjiang, China; 3 Qiqihar Medical University, Qiqihar, Heilongjiang, China

**Keywords:** antimicrobial sensitivity tests, bloodstream infection, colon tumor, *Leclercia adecarboxylata*, multidrug-resistant

## Abstract

*Leclercia adecarboxylata* is a motile, Gram-negative bacillus in the Enterobacteriaceae family and is ubiquitous in nature. In our study, we report a case of catheter-associated bloodstream infection caused by multidrug-resistant *L. adecarboxylata* in a colon tumor patient. The clinical symptom of the patient was high fever with chills. Initially, the patient was treated empirically with cefoperazone-sulbactam. Although cefoperazone-sulbactam was used for 4 days, the patient still had a fever. Then the bacterial cultures of the peripheral blood and catheter were positive for *L. adecarboxylata*. The treatment regimen was changed to meropenem. After the treatment, the patient’s clinical symptoms improved and the patient was discharged. Although *L. adecarboxylata* is regarded as a highly sensitive pathogen, it was a multidrug-resistant bacterium in our study. We recommend that the treatment of *L. adecarboxylata* should be based on antimicrobial sensitivity tests.

## 1 Introduction

*Leclercia adecarboxylata* is a motile, facultative-anaerobic, Gram-negative bacterium belonging to the Enterobacteriaceae family (1, 2). It is widely found in nature and can be isolated from food, water and other freshwater environments (3, 4). *Leclercia adecarboxylata* is an opportunistic pathogen that can cause septicemia, wound infection, urinary tract infection, posttraumatic polymicrobial infection, soft tissue infection, and peritonitis (5). It is worth noting that some patients with infection have fatal outcomes, including sepsis and death (6). Catheter-associated bloodstream infection has a very high incidence and mortality rate and is a healthcare-associated infection. It is usually caused by *Staphylococcus* (7). Although infections caused by opportunistic Gram-negative bacteria are rare, they have clinical significance.

To date, there have been no literature reports of catheter-associated bloodstream infections caused by multidrug-resistant *L. adecarboxylata* in colon tumor patients. In this study, we report a case of catheter-associated bloodstream infection caused by multidrug-resistant *L. adecarboxylata* after colon tumor surgery, providing valuable experience for clinical reference.

## 2 Case presentation

**Figure 1:**
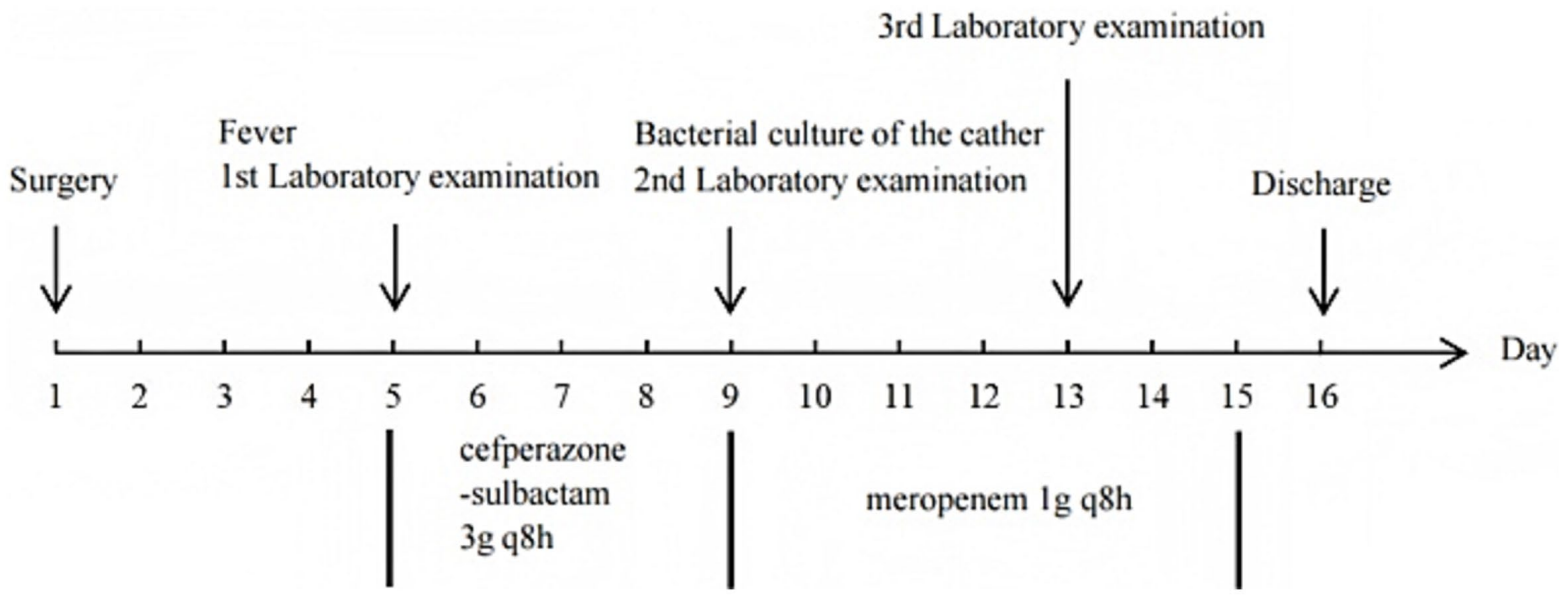
Clinical course timeline.

The patient was a 71-year-old man with a history of hypertension. The patient weighed 70 kg and his BMI was 22.49. He was admitted to the hospital because of colonic malignant tumor. The patient underwent radical resection of sigmoid carcinoma after admission. The operation was successful. The patient received parenteral nutrition through a central venous catheter (CVC). However, on postoperative day 4, the patient developed a fever (39.5 °C) and chills, with blood pressure of 150/75 mmHg, heart rate of 90 beats per minute, and the respiratory rate of 22 breaths per minute. Laboratory examination showed the white blood cell count (WBC) was 8.23 × 10^9^/L, the proportion of neutrophils was 88.1%, C-reactive protein (CRP) was 82.85 mg/L, and procalcitonin (PCT) was 2.08 ng/mL. The patient had no abdominal discomfort, no coughing or expectoration, and the drainage was normal. The surgical site was without exudate and the incision was healing well. Blood culture from venous was obtained and the patient was treated cefoperazone-sulbactam (3 g q8h iv). On postoperative day 5, no improvements were seen. The patient remained febrile. The patient’s blood pressure was 145/80 mmHg, the heart rate was 88 beats/min and the respiratory rate of 22 breaths per minute. The CVC was removed and sent for microbiological examination. On postoperative day 7, pathologic examination of the partial sigmoid colectomy specimen revealed a moderately differentiated adenocarcinoma invading the submucosa. All resection margins (proximal, distal, and inferior) were negative. No regional lymph node metastasis was identified (0/12 lymph nodes examined). On postoperative day 8, *L. adecarboxylata* was isolated from both peripheral blood and the CVC. The blood culture became positive after 8 h of incubation. 3 + *L. adecarboxylata* was isolated from the CVC. The antibiotic susceptibility test results were summarized in Table 1. The WBC was 6.24 × 10^9^/L, the proportion of neutrophils was 81.7%, CRP was 113.9 mg/L, and PCT was 5.18 ng/mL. The patient remained febrile with a temperature of 38.8 °C. Based on the clinical presentation of the patient and the results of bacterial culture, the antibiotic regimen was changed to meropenem (1 g q8h iv). On postoperative day 12, the patient was afebrile and clinically improved. The WBC was 4.86 × 10^9^/L, the proportion of neutrophils was 59.1%, CRP decreased to 9.49 mg/L, and PCT was 0.28 ng/mL (Table 2). The patient received meropenem for 7 days and was discharged on postoperative day 15. No adverse drug reactions were observed during the treatment (clinical course summarized in Figure 1).

**Table 1:** *Leclercia adecarboxylata* antibiograms.

Antimicrobial agents	MIC (ug/mL)	KB (mm)	Susceptibility
Cefazolin	≥64		Resistant^a^
Ampicillin	≥32		Resistant^a^
Ampicillin-sulbactam	≥32		Resistanta
Piperacillin-tazobactam	≤4		Susceptible^a^
Aztreonam	16		Resistant^a^
Ciprofloxacin	≥4		Resistant^a^
Levofloxacin	2		Resistant^a^
Ceftazidime	≤1		Susceptible^a^
Ceftriaxone	≥64		Resistant^a^
Ertapenem	≤0.5		Susceptible^a^
Imipenem	≤1		Susceptible^a^
Amikacin	≤2		Susceptible^a^
Gentamicin	≥16		Resistant^a^
Trimethoprim-sulfamethoxazole	≥320		Resistant^a^
Tobramycin	2		Susceptible^a^
Cefotetan	≤4		Susceptible^a^
Cefuroxime		6 mm	Resistant^b^
Cefoperazone sulbactam		17 mm	Intermediate^b^
Meropenem		29 mm	Susceptible^b^
Tigecycline		24 mm	Susceptible^b^

a Vitek2 automatic system using an AST GN card.

b Disk diffusion test based on Clinical and Laboratory Standards Institute (CLSI) recommendations.

**Table 2:** Blood test results.

Laboratory tests	Postoperative day 4	Postoperative day 8	Postoperative day 12
WBC (×10^9^/L)	8.23	6.24	4.86
Proportion of neutrophils (%)	88.1	81.7	59.1
CRP (mg/L)	82.85	113.9	9.49
PCT (ng/mL)	2.08	5.18	0.28

## 3 Discussion

*Leclercia adecarboxylata* was first described by Leclerc in 1962 as *Escherichia adecarboxylata*. It was renamed *L. adecarboxylata* in 1986 (8). It has been reported in the literature that *L. adecarboxylata* infection is associated with immunosuppression, catheters, wounds, malignant tumor (2). Bloodstream infection caused by *L. adecarboxylata* is usually associated with immunosuppression and the presence of catheters (9, 10). Most infected patients are immunosuppressed. In immunocompetent hosts, the pathogen is usually isolated as part of a polymicrobial infection (11). However, with improved of diagnostic methods, the detection rate in immunocompetent patients has increased (9). In immunocompetent patients with *L. adecarboxylata* infection, the identified risk factors include underlying disease, indwelling medical devices, the overuse of non-steroidal anti-inflammatory drugs and proton pump inhibitors (8, 12). In this case, the patient was diagnosed with colon tumor and received parenteral nutrition through the CVC. Both the colon tumor and the CVC were considered as risk factors for the patient.

*Leclercia adecarboxylata* is considered a relatively unserious pathogen due to its low virulence and high bacterial sensitivity (13). *Leclercia adecarboxylata* is sensitive to most antimicrobial agents, such as *β*-lactams, aminoglycosides, carbapenems, fluoroquinolones (14, 15). However, some multidrug-resistant strains causing catheter-associated bloodstream infections have been reported in two patients: a 47-year-old female with breast cancer and a 38-year-old female with gastric and duodenal diffuse large B-cell lymphoma (16, 17). The resistance mechanism of *L. adecarboxylata* to certain antimicrobial agents is drug exclusion by the bacterial outer membrane. The production of extended-spectrum *β*-lactamase enhances the reresistance of *L. adecarboxylata* (14). The drug sensitivity test of our case showed that *L. adecarboxylata* was a multidrug-resistant bacterium. *L. adecarboxylata* was resistant to quinolones, trimethoprim-sulfamethoxazole, gentamicin and most *β*-lactam antibiotics, but it was sensitive to piperacillin-tazobactam, ceftazidime, amikacin, tobramycin, cefotetan, tigecycline and all carbapenems.

There are no treatment guidelines and recommendations for *L. adecarboxylata*. Most isolates are sensitive to tested antibiotics (18). Spiegelhauer et al. (2) described several strains of *L. adecarboxylata* showing resistance to ampicillin and fosfomycin. Stock et al. reported the bacteria was naturally resistant to penicillin G, oxacillin, erythromycin, roxithromycin, clarithromycin, ketolides, lincosamides, dalfopristin-quinupristin, glycopeptides, rifampin, fusidic acid, inezolid and fosfomycin (19). Therefore, these antibiotics are not recommended for treatment. In our case, the patient was initially treated with cefoperazone-sulbactam, but unfortunately, the treatment was unsuccessful. The results of the drug sensitivity test showed that meropenem was sensitive. Meropenem rapidly penetrates most body fluids and tissues after intravenous administration. Renal excretion is the predominant pathway for meropenem elimination. Meropenem is a time-dependent antibiotic, and its efficacy relies on the percentage of time that free-drug concentrations are higher than the MIC (%T>MIC) (20). Based on the results of bacterial culture, the patient was subsequently treated with meropenem for 7 days and discharged.

The limitations of this study should be mentioned. Further prospective and well-designed clinical trials, especially with large samples, are needed to determine the treatment regimens for multidrug-resistant *L. adecarboxylata.*

## 4 Conclusion

We report a case of catheter-associated bloodstream infection caused by multidrug-resistant *L. adecarboxylata* that is treated successfully with meropenem. Bacterial culture should be carried out promptly during the treatment process. The results of antimicrobial sensitivity tests play a significant role in the treatment of multidrug-resistant *L. adecarboxylata*. When feasible, prompt removal of the catheter is a important measure for the treatment of catheter-associated bloodstream infection.

## Data Availability

The original contributions presented in the study are included in the article/supplementary material, further inquiries can be directed to the corresponding author.
